# Editorial: Interactions between proteins and biomacromolecules: Tools and applications (volume II)

**DOI:** 10.3389/fmolb.2022.1105486

**Published:** 2022-12-09

**Authors:** Fuming Zhang, Qunye Zhang, Lianli Chi

**Affiliations:** ^1^ Rensselaer Polytechnic Institute, Troy, NY, United States; ^2^ Qilu Hospital, Shandong University, Jinan, China; ^3^ Shandong University, Jinan, China

**Keywords:** interactome, biomacromolecule, protein, SARS-CoV-2, heparin

## 1 Introduction

The term “interactome” was first coined by Bernard Jacq in 1999 and means the whole set of molecular interactions in a cell. The interactome includes protein–protein interactions, protein-small molecule interactions, and protein–macromolecule (i.e., nucleic acids, lipids, carbohydrates, etc.) interactions. Throughout the past 2 decades, scientists have been trying to build up entire interactome networks of protein–protein interactions in different cells. It has been reported that a human cell may contain ∼130,000 binary interactions between proteins (Venkatesan et al., 2009). There are only 33,943 human protein–protein interactions listed on the interaction database, BioGRID (http://thebiogrid.org), suggesting that there is a significant need for further research efforts in this particular field. The interactome networks provide an insight into the mechanisms of cell functions under physiological and pathological conditions, which are critical for the discovery/development of the molecular targets for diagnostics or therapeutic applications (Bonetta, 2010).

For this second volume of this Research Topic, we have selected a series of articles that highlight technological advances for studying interactome. We hope these articles will provide useful information and protocols will be beneficial in facilitating the development of this important field.

## 2 Molecular docking in interactome

Molecular docking has been used in the characterization of interactions between proteins and biomacromolecules to determine the selectivity and binding affinity. As we know, protein–protein docking is one of the pivotal tools in computational biology, and with the development of computation technology (such as artificial intelligence, big data, super computer), docking analysis offers valuable information for fundamental studies of molecular interactions and provides a structural basis for drug design. In this Research Topic, Hassan et al. report on the synthesis and molecular docking analysis of novel heterocyclic-scaffold-based indole moiety as antimicrobial agents.

## 3 Interactome and biomarkers

A biomarker refers to a biomolecule found in bodily fluids, cells, or tissues that indicates a normal or abnormal disease condition. Biomarker discovery is critical to the development of clinical diagnostics and therapeutics. Interactome-based biomarker discovery can be achieved by Mass Spectrometry, NMR, SPR, and bioinformatics analysis.

Two articles in this special Research Topic examine biomarker discovery. In their study, Ding et al. report on nine genes that act as novel prognostic biomarkers based on integrated bioinformatics analyses of single-cell and bulk transcriptome data. Xian et al. discuss the development of a sensitive method of identifying specific marker peptides using liquid chromatography-tandem mass spectrometry with multiple reaction monitoring (LC-MS/MS-MRM) analysis.

## 4 Interactome and diseases

Neurodegenerative diseases, such as Alzheimer’s disease (AD), Parkinson’s disease (PD), and Huntington’s disease (HD) are serious dementia disorders. Interactome mapping offers a network of neurodegenerative-disease-related proteins, as Haenig et al. (2020) discuss, revealing an interactome network of ∼30,000 connections among ∼5,000 proteins involved in neurodegenerative diseases. There has been a significant research focus on the reduction of amyloid by inhibition of γ-secretase (GS) in AD drug discovery. Another study in this Research Topic by Eden et al. describes a novel approach to AD drug discovery by covalent fragment inhibition on intramembrane proteolysis. Zhou et al. report on the interaction between heparin and amyloid-beta peptide using heparin tetrasaccharides.

The COVID-19 pandemic is not over and will likely persist into the foreseeable future. Numerous mutations of SARS-CoV-2 have been identified, including Alpha, Beta, Gamma, Delta, and Omicron variants, with increased infectivity, immune evasion, and reinfection. Surface plasmon resonance (SPR) has been used in COVID-19-related basic studies and drug and vaccine development. The study by Gelbach et al. presents a detailed kinetic and structural analysis of the interactions of different SARS-CoV-2 S-protein RBDs with heparin, information that should be useful in anti-COVID-19 research.

Heparin-induced thrombocytopenia (HIT) is a heparin-related immune complication in heparin clinical applications. It is caused by antibodies directed to a complex of heparin and platelet factor 4 (PF4). Chen et al. reported a novel competitive biolayer interferometry (BLI) method to measure the binding affinity between heparin and PF4, which provided a deeper understanding of the heparin-PF4 interaction and a useful protocol for quality control in the production of heparin-related therapeutics.
